# “The Longest Way Round Is The Shortest Way Home”: An Overhaul of Surgical Ward Rounds

**DOI:** 10.1007/s00268-017-4267-1

**Published:** 2017-10-24

**Authors:** Kunal Shetty, Stephanie Xiu Wern Poo, Kumuthan Sriskandarajah, Michail Sideris, George Malietzis, Ara Darzi, Thanos Athanasiou

**Affiliations:** 1 Department of Surgery and Cancer, St Mary’s Hospital, Imperial College London, London, UK; 2 Queen Mary University London (QMUL), London, UK

## Abstract

**Background:**

Ward rounds, a keystone of hospital surgical practice, have recently been under the spotlight. Poor-quality ward rounds can lead to a greater number of adverse events, thereby cascading to an increased financial strain on our already burdened healthcare systems. Faced with mounting pressures from both outside and inside health organizations, concerted efforts are required to restore it back into prominence where it can no longer take a backseat to the other duties of a surgeon.

**Methods:**

The nucleus of this narrative review is derived from an extensive literature search on surgical ward rounds.

**Results:**

In this review, we focus on the need for reforms, current characteristics of surgical ward rounds, obstacles encountered by competing interests and proposed solutions in delivery of effective ward rounds that can meet with newly laid guidelines.

**Conclusion:**

Ward rounds should be standardized and prioritized to improve patient care.

## Introduction

In seventeenth-century Paris, the Board of Directors of Hôtel-Dieu Hospital in reply to the lobby posed by renowned French jurist Guillaume de Lamoignon on behalf of poor patients drew up its first code of conduct for its permanent clinicians. Concerns raised were that clinicians in favour of establishing private practices were neglecting poorer patients. In this code, the principle of daily ward rounds was established and, furthermore, mandated that a minimum of 2 h per day be spent in the examination of the hospital patients [1]. Ward round (WR) continues to be an integral aspect of hospital-based practice constituting a dynamic platform for members of a multidisciplinary team to integrate information from various resources and collectively make patient-centred decisions. Most importantly, it serves as a coherent communication channel, bedside from healthcare professionals to patients updating them of their daily progress. Its secondary purposes are education, fostering teamwork and leadership to name a few.

**Fig. 1:**
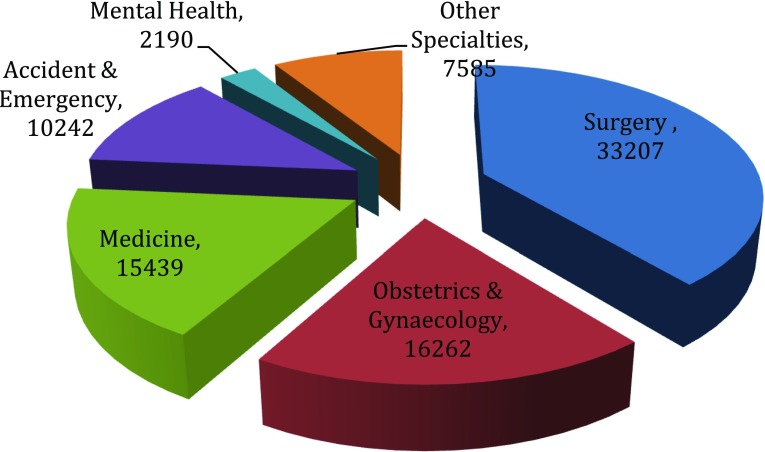
Total number of reported claims by specialty from April 1995 until 31 March 2013 excluding below excess claims handled by individual NHS trusts [4]

Today, in the twenty-first century, WRs are again in the spotlight, as part of a holistic re-evaluation of the delivery of hospital care following parliamentary, media and public scrutiny of failing National Health Service (NHS) Trusts. Annual figures released by the General Medical Council of UK reveal a steady rise in complaints against doctors to a record-breaking 10,347 complaints in 2012 [2]. Amongst the top three complaints were allegations of poor communication and a lack of respect, each of which has risen by 69 and 45%, respectively, in the year 2011 [3], and these have been shown to influence patient outcomes in terms of morbidity and mortality [4]. The 2013 NHS Litigation Authority report for clinical claims of negligence made in the last 18 years recorded the highest number against surgery (33,207) as illustrated in Fig. 1. Surprisingly, it was greater than the combined number of complaints received against Obstetrics and Gynaecology (16,262) and Medicine (15,439), which stood second and third, respectively. The burden inflicted by reported claims against surgery over this period has reached a staggering sum in excess of £3,198,241,000 (approximately £3.2 billion) [5].

These reports are not exclusive to UK surgical practice. 69.2% of general surgeons in the USA were sued at least once during their career which far exceeds their general internal medicine compatriots (34%) [6]. The Francis report of the Mid Staffordshire NHS Foundation Trust public inquiry unearthed that the failings of the investigated body were caused by poor communication to patients and that the surgical unit was dysfunctional [7]. Deteriorating doctor–patient relationship is recognized too within the medical community. A third of residents in a survey believed that their trainers did not represent good role models for doctor–patient relationships [8].

Therefore, improving the surgeon–patient relationship must regain focus in order to improve public trust and mitigate litigations with its associated financial burden. Efforts towards improving standardized surgical health care and outcomes have been skewed by the disproportionate interest in improving operative room (OR) processes [9, 10] over ward care. Unsurprisingly, adverse events arising from either the ward or OR have commonalities in that both are attributed to miscommunication, poor decision-making and inconsistency in performance [9–12].

Ward rounds [12, 13] along with operative skills [14] and handover [15] are identified as the three key areas in surgical care linked to improved patient outcomes. However, WRs often viewed as mundane tasks [16], in more than half the episodes are neglected by senior doctors and instead led by junior doctors [17] which is rarely the case in the OR or out-patient clinic. Recent evidence suggests that discrepancies in short-term surgical outcomes, for example mortality, between hospitals are reliant on the quality of post-operative ward care [18]. Hospitals that possess an infrastructure capable of not only detecting but also escalating unwell patients have demonstrated lower mortality rates [19, 20].

In addition, patients are at increased risk of death if either admitted as an emergency during the weekend when hospitals are comparatively understaffed or if they underwent an elective surgical procedure at the end of the working week [21–23]. Lack of a senior clinician-led WR on weekends imposes patients into “hibernation” ultimately leading to delays in investigations, diagnosis, treatment and discharge from the hospital [24]. On the contrary, a consultant-led WR twice a day was shown to drastically reduce in-patient stay by half (10.4–5.3 days; *p* < 0.01) with no effect on the readmission rate [17]. Recognizing the link between on-site presence of senior doctors to lower mortality risk of patients admitted during out of hours, the steering group of the Academy of Medical Royal Colleges led by the President of the Royal College of Surgeons of England suggested a daily consultant presence care system. The first proposed standard recommends that all in-patients should be provided consultant-led reviews, unless it has been determined that this would not affect the patient’s care pathway [24]. Recognizing the variability in the quality and frequency of consultant-led ward rounds, the Royal College of Surgeons of Edinburgh [25] have welcomed the joint initiative by the Royal College of Physicians and Royal College of Nurses in establishing “best practice principles” for medical ward rounds [26].

The objectives of this review are to outline the quantitative and qualitative factors of surgical ward rounds (SWRs), hurdles encountered and current guidelines.

## Methods

### Search strategy

Studies were identified by an electronic search using MEDLINE, EMBASE and Cochrane Databases (1 January 1975–1 September 2016) with the following keywords simultaneously: “ward rounds, surgery, surgical visits”. Studies were selected for further analysis based on careful reading of the available abstracts. Additional studies were obtained by reference crosschecking.

### Inclusion and exclusion criteria

The following inclusion criteria were used: all patients were treated for a surgical condition and a defined ward round process was documented; only fully published articles were eligible for inclusion to reduce heterogeneity. No language restrictions were applied. Two reviewers (GM and KS) independently assessed studies for inclusion and completed data extraction into an electronic database, with disagreements resolved by consensus with a third reviewer (TA).

Results and discussion were presented after data were pooled into eight main categories (Surgical Ward Round—Definitions, Mapping Surgical Ward Rounds, Membership in Surgical Ward Rounds, Communication and Leadership issues, Simulation and Education, Record Keeping and Need for Development).

## Results and discussion

### Surgical ward round

SWRs possess unique traits differentiating it from other medical specialty ward rounds. Firstly, consultant surgeons have to divide their weekly clinical commitments between in-patient ward care (elective and emergency), out-patient clinics, operative theatres (elective and emergency) and administrative duties. Secondly, surgical patients differ from medical patients in that they usually transition from a surgical ward to an OR with an option of ICU before returning to the surgical ward. In terms of stepping up to the OR and step down back to the ward, their ward care is complex requiring judicious monitoring of a number of invasive adjuncts such as airways, drains, catheters, pumps, stomas, arterial lines, peripheral and central venous lines. Enhanced recovery programmes for major elective surgery have created a framework for multidisciplinary pre-operative and post-operative care which have ultimately shown proven benefits in terms of decreasing hospital stay and lowering complication rates [27]. However, the lack of similar recovery programmes in emergency surgery can be supplanted by structured and standardized SWRs, which direct a multidisciplinary effort in targeting the various aspects involved in the recovery of a surgical patient.

### Mapping surgical ward rounds

An analysis of a surgeon’s work pattern revealed that on an average workday of 9 h 26 min, the surgeon spends 2 h 3 min on documentation and administration, 1 h 47 min on operative procedures, 1 h 43 min on internal communication and just 48 min on ward rounds [28]. On further inspection, the average time spent per patient at the bedside was under 2 min 30 s [29] which is less than a third of the recommended average of 9–10 min to be spent per patient as proposed by the Royal College of Physicians guidelines [26]. If it were to be enforced, a SWR for 20 patients would last approximately 3½ h. Understandably, the recommended average time spent per patient on a routine medical ward round is probably calculated based on the time required on a medical ward round whilst using a ward round checklist [30] and need not apply to SWRs.

However, it may be safe to infer that the observed average time spent per patient (2½ min) on a SWR is insufficient to address all relevant issues. This might be attributed to the pressures faced by the surgeon from numerous commitments where SWRs seldom take precedence. Another observation on both medical and surgical ward rounds was that the average time spent on patients admitted in outlier wards was greater [29, 30] yet a comprehensive multi-centred study revealed that nearly a quarter (22.7%) of admitted surgical patients were located at outlier wards [17].

### Membership in surgical ward rounds

**Fig. 2:**
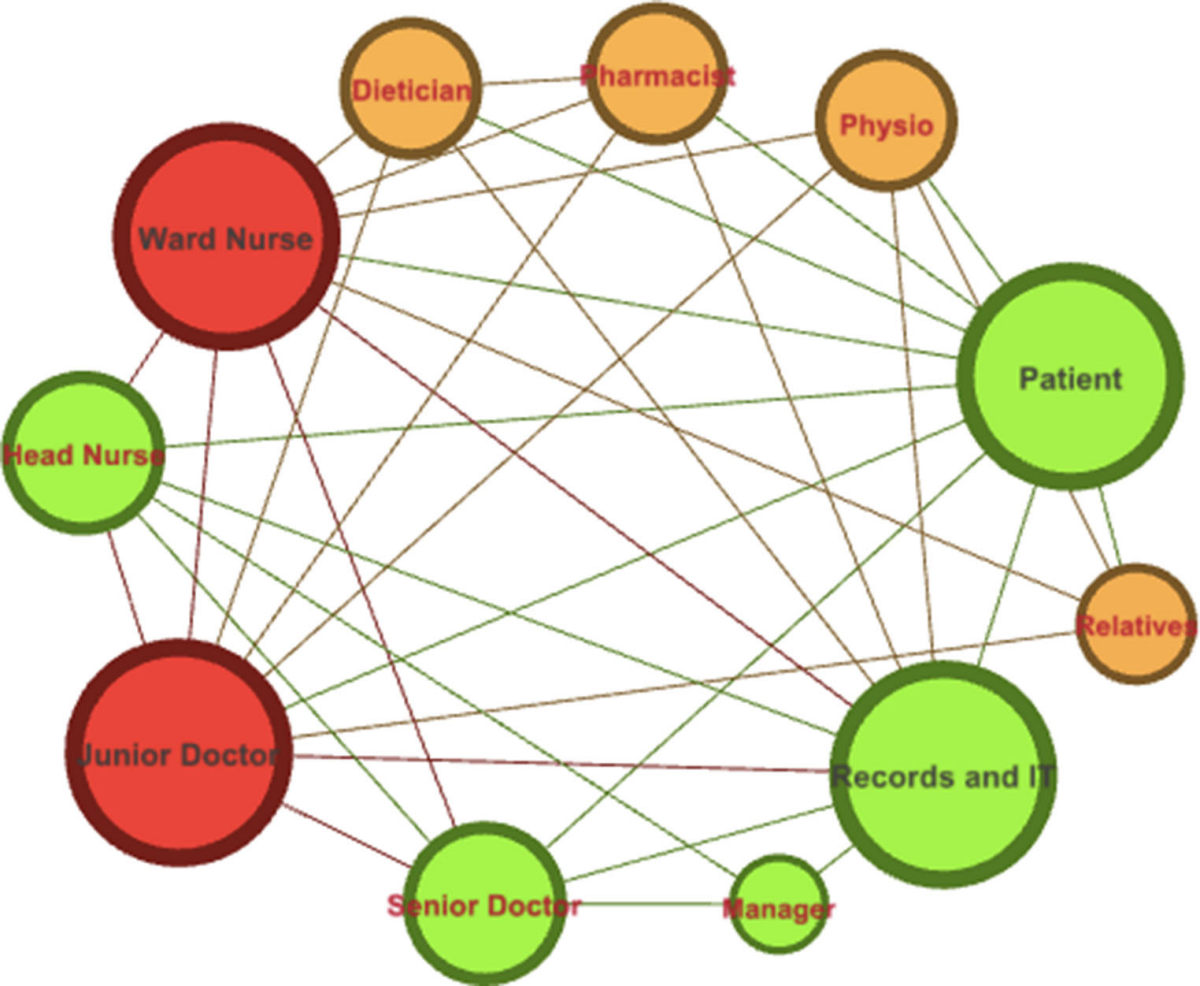
Illustration of the complex network between patients and ward round team members. “Stable” members of the SWR are denoted in green and “unstable” in red. An increase in the number of interactions by a member of the surgical ward round team corresponds to an increase in the size of its representative circle

Daily WRs are usually led by a consultant surgeon and in his absence led by a registrar or senior resident. In addition to the aforementioned, the remaining members of the SWR comprise of junior doctors, patient’s assigned nurse, allied health professionals such as physiotherapists, pharmacists, dieticians and occupational therapists. Dependent on patients’ individual characteristics and ward, other specialists such as nurses with specialty interests (e.g. tissue viability, diabetes care and stoma care), consultant intensivists and microbiologist may also be present. Although each member plays a specific role within the team, these roles are mutually complementary sharing collective responsibility for delivery of ward care. Although surgical conditions in the last 50 years have not become more complex, surgical interventions have led to an expansion in the SWR team which in turn has resulted to a complex network of interactions between members as illustrated in Fig. 2.

However, the stability of ward round members is affected by the multiple changeovers of junior doctors and ward nurses due to restriction in working hours, on-call and educational commitments ultimately threatening continuity of patient care. This lack of continuity amongst frontline staff has ushered the need for improved process for handover [15]. Junior doctors participating in SWRs are largely composed of training and non-training grade doctors who spend between 4 and 6 months within a surgical firm. During the initial period of their tenure, they are rapidly required to grasp the SWR practices and preferences of the local hospital, unit and consultant. This requires a settling period to adapt to local practices ranging from a few weeks to months. By standardizing ward round protocols with room for minor modifications, it would not only decrease the effort in incorporating fresh junior doctors but also create a transparent culture of the team’s expectations and best practice. Completing the complex SWR network are patient relatives and documentation whether it is in paper (hard copy) or electronic (digital). Inclusion of patient relatives is critical as not only can they be a source of key information but they also play a major role in decision-making, creating a support system for the patient and adherence to therapy.

The final “member” of the network or the team, and probably its most stable one, is the patient’s record. Critically, it serves as a communication channel between all team members who have not simultaneously reviewed the patient on the SWR. However, the quality of record keeping is very much user dependent and every effort must be made to conform to the guidelines laid by the Royal College of Surgeons of England [31].

### From communication to collaboration

Advantages of effective collaboration include: it can improve quality of health care and patient safety and also reduce workload issues that might induce burnouts amongst healthcare professionals. Similarly, failures in team work and communication are well-recognized threats to patient safety leading to adverse events [32]. Larger-sized teams and altering team dynamics are considered to be important factors, which can cause breakdown in continuity of care [33]. Core members of a SWR, namely doctors and nurses, have shown inadequate direct communication as in only 44% of SWRs [17] nurses are present. Instead, patient’s notes form the main channel of communication between these professionals, who disagree on priorities for patient care [34, 35].

Some of the barriers that may have contributed to poor collaboration include professional hierarchy, discrepancies in perception of effective communication and teamwork. Nurses’ perception of good collaboration is one that has their input acknowledged and respected, whilst that of physicians is of nurses that “follow instructions” and “anticipate needs” [36, 37]. These differences in ideology and “political power” may have deep roots in medical and nursing training [36].

Therefore, WRs should be targeted for fostering effective collaboration and teamwork as drivers for good clinical care. Current barriers to multidisciplinary WRs (MDWRs) are that they are inadequately prioritized by all HCPs, variable work shift patterns, lack of concerted efforts, frequent change in lead consultant, specialty, wards and poor handovers to name a few [25]. Doctors feel that nursing support was by chance and not routine, whilst nurses believe that the presence of senior doctors on ward rounds is unpredictable, irregular and therefore they are unavailable to participate leading to assumption that consistent MDWRs in UK hospital [38] are futile.

The unpredictable and irregular availability of the members of the multidisciplinary team during ward rounds pose a challenge to share patient information and provide high-quality care. Until recently, pagers have been the sole means of communication between healthcare professionals when physical means are not possible. They are reliable, however, suboptimal and inefficient in that they deliver one-way communication, which result in workflow and ward round disruptions.

Addressing these challenges, recommendations are required of making WRs a priority for all HCPs, by dedicating a set time for MDWRs, identification of staffing issues before the WR, assignment of individual roles to each member to improve engagement, improved communication technologies between healthcare professionals and the presence of a senior nurse at every bedside review [26]. Considering that 78% of SWRs commence before 9 a.m. [17], with re-arrangement of staffing priorities and levels, a co-ordinated well-attended SWR is feasible. When implemented, MDWRs, structured interdisciplinary rounds and daily goal setting has shown to improve communication, nurses’ ratings of teamwork and patient outcomes [39–41].

### Need for leadership

**Fig. 3:**
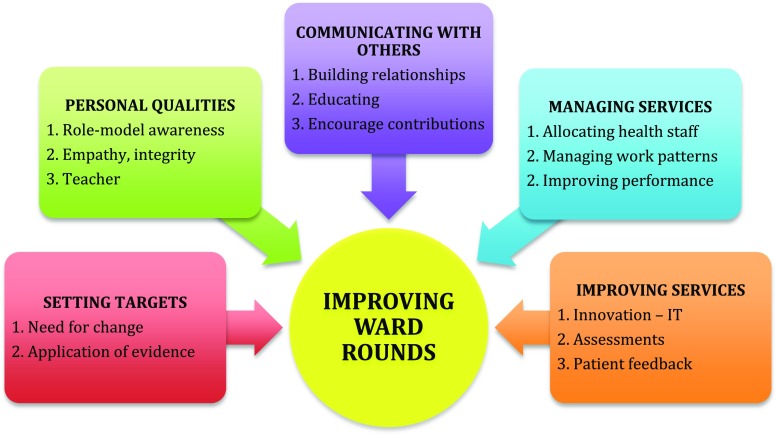
Strategy to lead improvements in surgical ward rounds, adapted from the NHS leadership academy model

Increasingly surgeons are required to demonstrate leadership qualities in the context of their own immediate team, across teams, across services or the entire organization [42]. To improve the current format and to bring SWRs back into prominence, it will require vision, motivation, professionalism, communication, teamwork, resilience, networking, innovation and business acumen to name a few as illustrated in Fig. 3. These ingredients are some of the necessary leadership attributes [43] a surgeon must demonstrate in order to adapt to the major reformations transforming the NHS whereby as an individual and a community we could continue to exercise our influence in improving patient care. Unlike the past, surgical leaders can no longer rely on solely their reputations but are now required to demonstrate their understanding of the new business models of healthcare delivery, ability to tackle adaptive challenges, resolve conflict, demonstrate success in not only improving themselves but also others and emotional competence [44]. Instead of the belief that to lead is in one’s DNA, the drive of the NHS leadership academy is to forge it by introducing a framework which constitutes a part of undergraduate and postgraduate medical curriculum and education [45].

### WR the soul of patient-centred care

In the aftermath of the Francis report, the current UK health minister (Jeremy Hunt) in a recent speech stated initiatives must take place to end fragmented care and the culture of treating patients as a “critical business”. Instead, patients should be treated as people with compassion and empathy [46]. The report “High quality care for all: NHS Next Stage Review final report” has reoriented our focus on treatment of patients with compassion, dignity and respect [47]. The emphasis is on publishing healthcare data capturing patients’ own views on the success of their treatment and the quality of their experiences in addition to measures of safety and clinical outcomes. Patients’ experience and views on the ward round can provide valuable feedback and opportunity for improvements in SWR practices.

Although limited studies are available capturing patients’ perception of SWR, they are found to be overall positive even in the grand round format. Patients when provided prior explanation about the purpose of the SWR were found to have significantly better understanding of the process and therefore improve engagement [48, 49].

### The role of education

Educating trainees is a privilege and an obligation of the medical profession as recited in the Hippocratic Oath. Surgical management principles have become increasing complex with the introduction of minimally invasive techniques, interventional procedures and allied therapies. Yet trainees face severe challenges in surgical skills acquirement brought about by working hour restrictions. For instance, post-take ward rounds (PTWRs) serve as vital educational experiences to receive feedback on management plans of newly admitted patients from the admitting consultant (trainer). Interestingly, the provisional diagnosis was frequently altered in 27% of cases at PTWRs. Sadly trainees who miss PTWRs due to working hour restrictions fail to learn from their mistakes [50].

To maximize the educational ability of a WR, the leader should make prior plans that account for time spent, educational needs, assign the role of the teacher, create an educational environment and finally assess if these aims have been achieved [51]. Medical educational reforms have tried to separate from traditional Halstedian apprenticeship models, but some fundamental aspects of surgical training are still mired in it. Clinical teachers serve as role models to trainees who pattern themselves consciously or unconsciously on their attributes [52]. Excellent role models in medical education were associated with greater time spent on teaching, emphasis on doctor–patient relationship and focusing on the psychosocial aspects of medicine [53]. Role modelling can be improved both at a personal and at an institutional level. At a personal level, it requites conscious self-recognition of the importance in it, allocating sufficient time to teach, self-reflection and participation in team building exercises [52].

### How can learning be assessed?

Current surgical trainees in the UK are required to maintain an online digital record of their work-based assessments to demonstrate competency in training. In order for improvements to occur, it must be measurable. Assessments in the form of clinical examination skills and case-based discussions offered online via the Intercollegiate Surgical Curriculum Project [54] permit surgical trainees to continuously demonstrate knowledge, clinical skills, decision-making ability and non-technical skills that are necessary for proficient SWRs. Deficiencies in existing tools are that they insufficiently focus the assessment on skills necessary for WRs. Modified tools specifically designed to assess SWRs based on criteria set by checklists have been designed and piloted for the assessment of surgical trainees [55]. Toolkits have also been developed in the form of checklists to capture clinical skills, team interaction skills and doctor–patient interactions during a SWR [56]. Recently, a Quality of Information Transfer Tool has been developed and validated to further improve and directly combat the communication failures within WRT members and poor escalation of care, which previous tools such as SBAR have failed to address [57]. This objective training tool incorporates key clinical information and a solid presentation structure, whilst assessing the efficacy of communication skills training during escalation of care.

### Simulating surgical ward rounds

Evaluation of doctor’s interaction processes with a patient in a simulated environment has been used for almost a decade in medical licensing examinations [58]. In surgery, it was originally introduced to improve operative skills in a controlled environment before transferring it to the OR [59]. Today simulation is increasingly used to improve non-technical skills of surgeons amongst which one of them is to undertake efficient SWRs. High fidelity virtual world simulation can create an easily accessible online portal to teach and assess surgical ward care processes [60]. Alternatively, real-time highly immersive simulated ward environments have been created and validated to assess SWRs. Simulated studies have revealed that junior surgical residents when leading a SWR manage lesser issues integral to a SWR, thereby committing greater adverse events when compared to senior residents [61]. Initial expenses in creation of a four-bedded simulation ward are a moderate amount of £5000. Additional running costs of a simulated ward round involve hiring of actors at £25 per hour comparable to expenses of conducting a simulated operating suite [62]. Despite its costs, the benefits are that it allows repeated practice in a safe and controlled environment free from compromising the well-being of real patients [56].

### Checklists

Checklists are observed to improve adherence to care processes in simulated surgical scenarios [9] and have demonstrated profound impact on reducing adverse events in the OR [10] leading to the widespread implementation of the WHO surgical safety checklist in six different languages. In comparison, the introduction of ward round checklists has received less enthusiasm despite 53–70% of surgical errors occurring outside the OR [63–66] and that the quality of SWRs is observed to be highly variable where lower-quality SWRs are linked to a greater number of preventable complications [12].

**Fig. 4:**
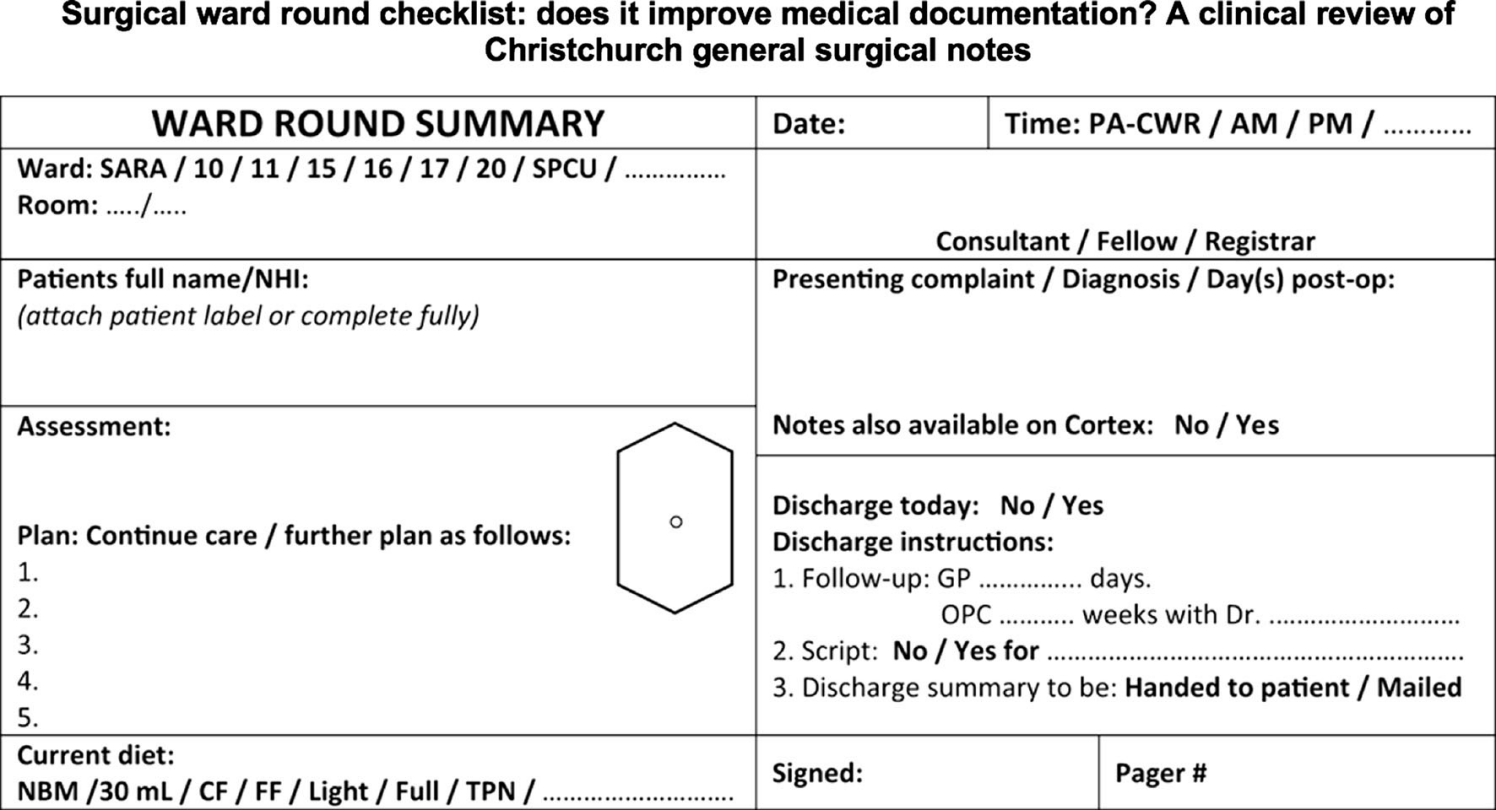
Modified example proforma sticker, which are pre-populated in order to serve as prompts and improve documentation during surgical ward round

**Fig. 5:**
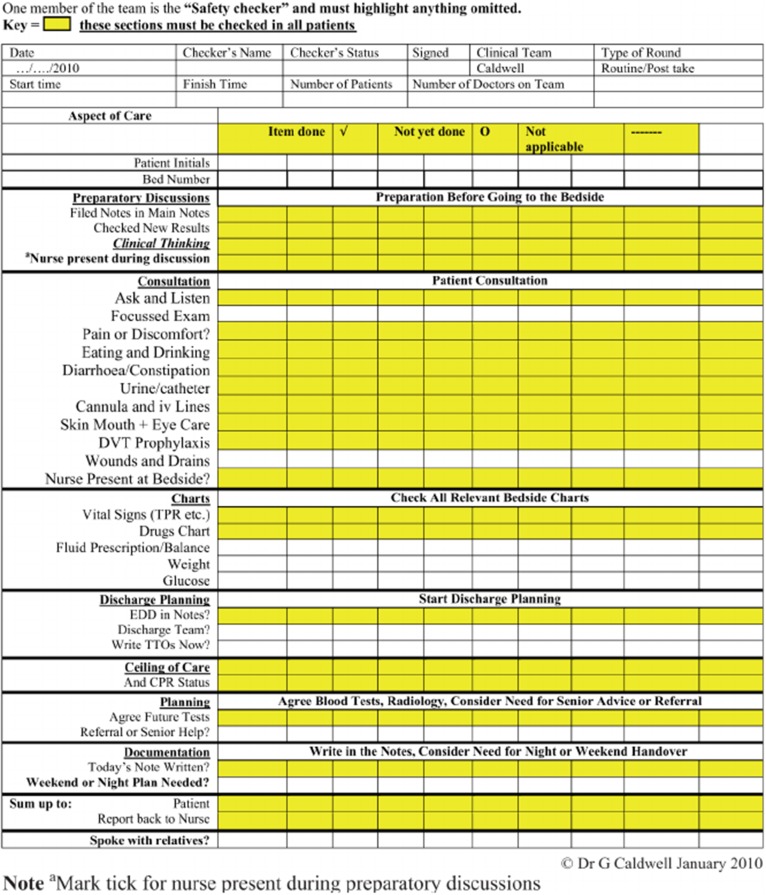
Ward round checklist based on Caldwell “Considerative Checklist”

Addressing the need for standardizing ward rounds a comprehensive ward safety checklist has recently been developed [38]. It consists of three universal phases, namely introduction (phase one), timeout (phase two) and action (phase three) which in turn address 20 points. A provision is made to cover extra issues relevant to one’s specialty between phases one and two. Implementation of the ward safety checklist in an acute surgical unit has shown to improve consistency in completeness of SWR, but associated benefits and average time taken per patient were not measured [67]. Comprehensive WR checklists introduced in the past, when implemented, have been found to take on average 12 min per patient for completion [30]. A recent study by Alamri et al. [68] proposed a modified proforma in order to improve documentation as well as serve as important prompts during fast-paced surgical ward rounds (Fig. 4). Key ingredients in surgical checklists include: patient details, members of the multidisciplinary team, bedside patient consultation, history and examination findings, review of drains/tubes/lines, charts and medical investigations, documentation, estimated discharge date and presence of relatives/relevant discussions (Fig. 5) [68, 69]. Indeed with advances in electronic patient records, surgical ward round templates can be introduced to ensure standardization and optimization of patient care—variations of these proformas can then be adjusted to suit the specifics in each specialty. Caution must be taken in designing extensive checklists as their acceptance can be affected if prescriptive and time-consuming. The successful dissemination of the WHO safe surgery checklist over the more comprehensive SURPASS checklist hinged on its succinctness (22 points vs 90 points) and requires approximately 2 min for completion. Other disadvantages of checklists are: it requires a designated member of the WRT to be assigned the checker’s role, shifts the focus of attention away from the patient to the checklist, limits the leader’s intellectual autonomy and saturates the documentary process.

Benefits of using checklists can be appreciated as an “aide memoire” to residents in the early phases of their training who are yet to form a structured method of leading SWRs. It also can be incorporated into the educational curriculum of both undergraduate and postgraduate students. Additionally, checklists can be used as assessment tools for revalidation, undergraduate and postgraduate examinations.

### Record keeping and use of technology

Good surgical practice recommends that surgeons must diligently at all times maintain *legible, complete and contemporaneous* medical records [31]. Poor documentation has been associated with poor patient care and increased rates of adverse events [70]. In the current climate of increased shift work patterns brought about EWTD, the absence of one or more members of the WRT and rising medico-legal claims, maintenance of records is ever more important for continuity of care, communication between WRT members and as a medico-legal record. Implementation of structured proformas could improve the standards of record keeping in the UK that are found to be highly variable and often deficient [71, 72].

Healthcare professionals have increasingly moved away from traditional paper records to embracing technological advances that permit maintenance, sharing and storage of records digitally in the form of *electronic patient records* [73]. However, cautions must be taken before rushing to adopt EPR in its current format as it has been observed that these devices are obtrusive thereby altering team dynamics [73] and shifts the focus of attention from the patient to the electronic device resulting in negative patient experiences [74]. These deficiencies can be overcome by creating transparent, miniaturized electronic gadgets and training HCPs to alter their unconscious behaviour created by the introduction of electronic aids [74]. Efforts in development and incorporation of EPR are worthwhile as the benefits from it are limitless. It would allow for multiple users to simultaneously, remotely access in real-time up-to-date medical records and investigations at their fingertips. Other benefits include cost and error reduction, process automation as well as enhanced clinical documentation and decision support which aims to improve patient health care. Automatic error detection systems could potentially be developed to prevent adverse events. For example, drug interactions for prescribed drugs could be detected using intelligent systems, automatically alerting the clinician [74] or an inappropriately requested investigation (X-ray) for the wrong limb could be detected. In addition, mobile devices have been shown to enhance patient education in the hospital setting [75].

However, despite quicker access to hospital information and improved methods for information sharing, there are unintentional and feared side effects of increasing reliance on technology/e-health care, which include alert desensitization, reduction in clinical acumen and in the ward round setting, reduced patient focus and face-to-face engagement. In addition, some healthcare professionals have found the use of “computer on wheels” practically difficult. Baysari et al. [75] explored the impact of iPads, a relatively more convenient portable device on doctor–patient relationship: majority of patients did not think that iPad use impacted their engagement with doctors on rounds. Indeed, patients suggested that being offered choices and results of investigations in real time through the use of iPads helped them to feel more engaged in their care process [75]. Nonetheless, the impact of e-health records on ward rounds should be regularly audited to ensure patient care is not compromised from the increasing use of technology.

The OpenNotes trial is an ongoing project which allows patients to view personal medical records live and this may become a future norm to encourage their involvement in medical decisions and care [76]. In this way, increasingly tech-and medical literate patients may be able to feed back in “real-time” clinical information, e.g. symptoms or any concerns to the clinical team [77].

Telecommunication advances have empowered off-site surgeons to conduct and participate in SWRs whereby the surgeon uses a joystick to remotely navigate a five-foot-tall robotic system on wheels named RP-7i [78]. Interaction in this form has been found to be acceptable by patients [79].

In order to improve handover and transfer of information, current communication technologies must be updated. Increasingly, pagers have been used in parallel with mobile devices as a means for interprofessional communication between healthcare teams. Mobile applications, particularly Whatsapp, are now widely popular and cost-effective communication tools amongst HCPs. Smartphones provide multiple communication modalities within teams in and outside of hospital, and these have been shown to provide greater satisfaction and perception of efficacy [80–83]. A randomized controlled trial comparing a new mobile phone app, Hark with usual paging systems, demonstrated superiority of the app in terms of quality of information transfer, rated highly effective and had better response time by users with no delays in patient care [84, 85]. The main disadvantages of mobile phone communication are need for a phone signal, Wi-fi or Internet network; concerns regarding security of information transfer; and potential for misunderstandings due to the lack of non-verbal communication [86]. However, these can be overcome with the development of a robust user-informed guide for the implementation of application-based communication system, taking into account the concerns regarding confidentiality and involving multiple stakeholders [87].

### Surgeon’s dress code

The doctor–patient relationship, and thus the ward round interaction, is influenced by the patient’s perception of the doctor. Traditionally, the doctor’s attire has reinforced stereotypes of authority and competence. In 2007, the Department of Health burgeoned by public concerns on hospital acquired infections recommended that doctors adopt a “bare below the elbow policy”, thereby refraining from wearing long-sleeved shirts, wrist watches, long ties and traditional white coats [88]. When patients opinions on a surgeons attire were sought, majority of them indicated no strong preference for a certain type of attire and it did not significantly affect their perception of safety from infection or the type of care received [89, 90].

Surgical patients are at risk of acquiring infections due to their increasing age and size (obesity), comorbidities (diabetics), medication (immunosuppressants) and presence of wounds. Consequences of acquired infections can be catastrophic leading to lengthier stay, additional investigations, procedures and antibiotic therapy that ultimately may contribute to an increased risk of mortality. Despite inconclusive evidence, every precaution should be taken to minimize transmission of infections by simple and effective measures even if established current evidence is inconclusive and therefore it is advocated a “bare below elbows” policy.

### Improving the quality of surgical ward rounds

The greatest gift from a doctor to his patient is time [4]. Adequate time spent on addressing most if not all of patients’ concerns, ensure clear management plans, regular updates and providing empathy when needed will have everlasting impression on whom we treat. Evidence suggests that the current state of SWRs is inadequate reflected by the time spent per patient and the mounting number of legal lawsuits against surgeons. SWRs must imminently regain one of the primary focuses of patient-centred hospital care [16] and no longer take a back seat to other responsibilities of the surgeon. Recent guidelines by the Royal Colleges too have stressed the importance of reforming ward care, yet there is a lack of clear strategy in tackling this issue [24, 26].

**Fig. 6:**
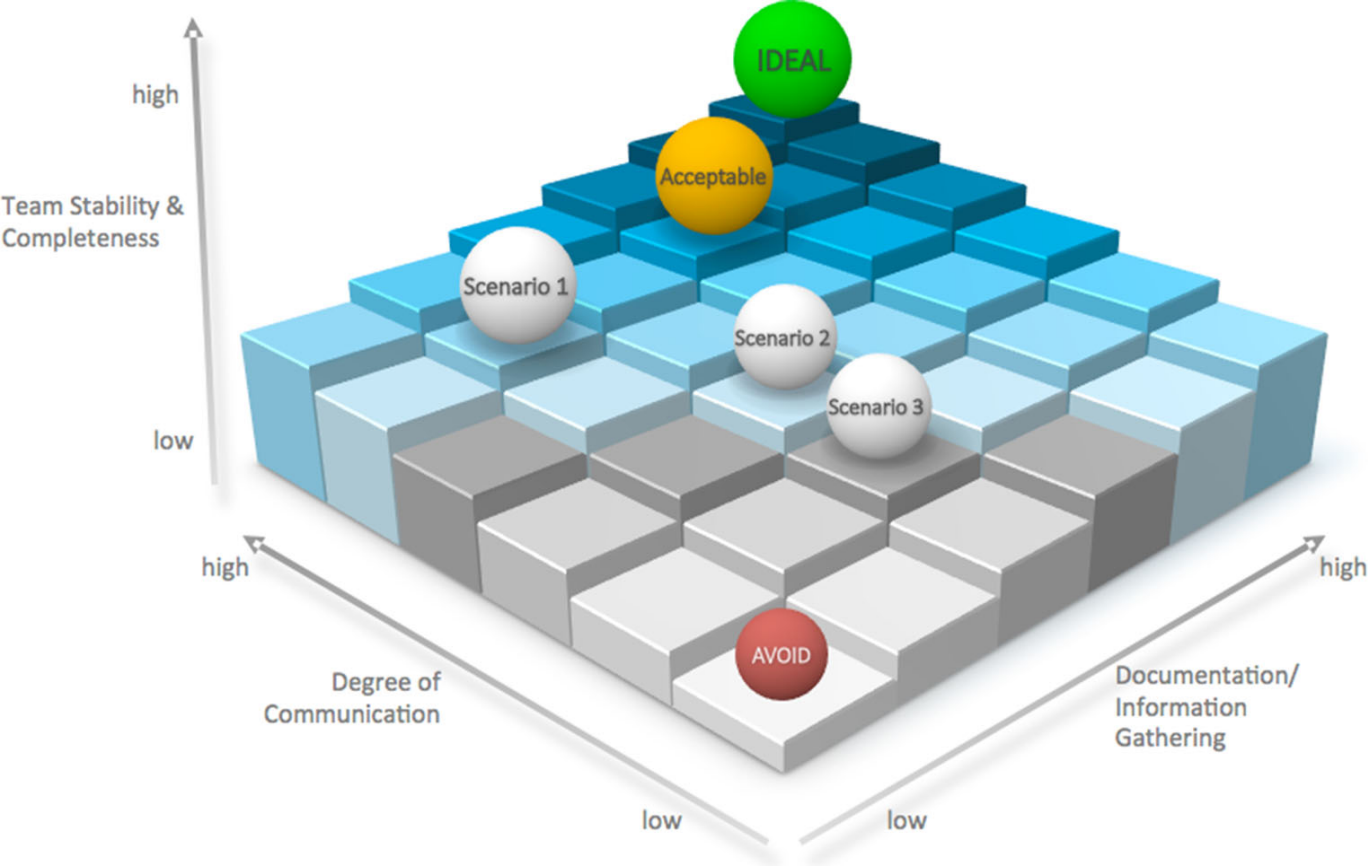
A proposed model to assess quality of surgical ward rounds. Team stability, degree of communication and documentation are key requisites of a satisfactory surgical ward round. Inadequate levels of any of these three components may result into a scenario of poor-quality SWR which must be avoided

Quality metrics must be established to measure the quality of current SWRs in order to find areas for improvement. In this review, we identify three essential factors for an adequate surgical ward round as illustrated in Fig. 6. They are firstly team stability and its completeness, secondly the degree of communication and thirdly information gathering or documentation. All three of these factors must be maintained as suitable levels. A failure to do so will result in poor-quality surgical ward rounds ultimately threatening patient safety.

A review on shift patterns and staffing levels is required at an organizational and local level to facilitate multidisciplinary ward rounds. Surgeon’s work schedules must have flexibility to accommodate post-take ward rounds where ample time is spent per patient. Consultant surgeon’s job plans require reconfiguration to focus adequate time on conducting quality SWRs. Currently available forecasting software tools are capable of predicting the number of emergency attendees, admissions, discharges and the time spent per operative procedure in OR, based on which information hospital managers can create weekly schedules. This information should also be used provide matching allocated clinical hours dedicated for SWRs.

## Conclusion

Surgical ward rounds, operative care and handover are linked to patient outcomes. Initiatives should be taken by the hospital management to admit patients at almost all times to their assigned specialty wards to prevent unnecessary time wasted on already hard-pressed surgeons. Modern technology may be useful adjuncts in facilitating the seamlessness of the workflow of a SWR and allowing access for surgeons from remote locations to review their patients. However, steps must be taken to make existing technologies unobtrusive. SWRs should be standardized and prioritized as educational opportunities whereby roles can be switched to allow more junior members of the team to lead the SWR. Checklists and simulation have value for the inexperienced clinician, but the verdict on its universal applicability is still pending.

## Abbreviations

EWTD: European Working Time Directive
HCP: Healthcare professionals
MDWR: Multidisciplinary ward round
NHS: National Health Trust
OR: Operating room
PTWR: Post-take ward round
SWR: Surgical ward round
WR: Ward round
WRT: Ward round team
